# Deficient Vitamin E Uptake During Development Impairs Neural Tube Closure in Mice Lacking Lipoprotein Receptor SR-BI

**DOI:** 10.1038/s41598-017-05422-w

**Published:** 2017-07-12

**Authors:** Nicolás Santander, Carlos Lizama, María José Parga, Alonso Quiroz, Druso Pérez, Guadalupe Echeverría, Lorena Ulloa, Verónica Palma, Attilio Rigotti, Dolores Busso

**Affiliations:** 1 Department of Nutrition, Diabetes and Metabolism, School of Medicine, Pontificia Universidad Católica de Chile, Santiago, Chile; 2 Center of Molecular Nutrition and Chronic Diseases, School of Medicine, Pontificia Universidad Católica de Chile, Santiago, Chile; 3 Cardiovascular Research Institute, University of California, San Francisco, California USA; 4 Laboratory of Stem Cell and Developmental Biology, Faculty of Sciences, Universidad de Chile, Santiago, Chile

## Abstract

SR-BI is the main receptor for high density lipoproteins (HDL) and mediates the bidirectional transport of lipids, such as cholesterol and vitamin E, between these particles and cells. During early development, SR-BI is expressed in extraembryonic tissue, specifically in trophoblast giant cells in the parietal yolk sac. We previously showed that approximately 50% of SR-BI^−/−^ embryos fail to close the anterior neural tube and develop exencephaly, a perinatal lethal condition. Here, we evaluated the role of SR-BI in embryonic vitamin E uptake during murine neural tube closure. Our results showed that SR-BI^−/−^ embryos had a very low vitamin E content in comparison to SR-BI^+/+^ embryos. Whereas SR-BI^−/−^ embryos with closed neural tubes (nSR-BI^−/−^) had high levels of reactive oxygen species (ROS), intermediate ROS levels between SR-BI^+/+^ and nSR-BI^−/−^ embryos were detected in SR-BI^−/−^ with NTD (NTD SR-BI^−/−^). Reduced expression of Pax3, Alx1 and Alx3 genes was found in NTD SR-BI^−/−^ embryos. Maternal α-tocopherol dietary supplementation prevented NTD almost completely (from 54% to 2%, p < 0.001) in SR-BI^−/−^ embryos and normalized ROS and gene expression levels. In sum, our results suggest the involvement of SR-BI in the maternal provision of embryonic vitamin E to the mouse embryo during neural tube closure.

## Introduction

Scavenger Receptor Class B type I (SR-BI) is the main receptor for high density lipoproteins (HDL), and numerous studies have described its role in mediating the bidirectional transport of lipids between these lipoproteins and cells^1^. In the liver, SR-BI is involved in the uptake of cholesterol from HDL and its excretion in bile, the final step in reverse cholesterol transport. SR-BI also participates in the uptake of cholesterol in steroidogenic tissues, such as the adrenal glands and ovaries, to be used as a substrate for steroid hormone synthesis^2^. Important information on the roles of SR-BI other than in cholesterol homeostasis and cholesterol provision for steroidogenesis, such as platelet aggregation, erythrocyte maturation and oocyte meiosis, has been generated from the SR-BI knock out (SR-BI^−/−^) mouse since it was generated almost two decades ago^3^.

In generating SR-BI^−/−^ mice via heterozygous intercrosses, researchers noted that the proportion of weaned homozygous null mice was half that expected by the Mendelian ratio^3^. This evidence, together with the fact that SR-BI is present in murine trophoblasts involved in maternal-foetal nutrient exchange at different stages of gestation^4^, led researchers to postulate that this HDL receptor might be involved in embryonic development. We recently showed that nearly 50% of SR-BI^−/−^ embryos fail to close the anterior neural tube and develop cranial NTD and exencephaly^5^, leading to perinatal death, which explains the deviation from the Mendelian ratio previously reported in weaned SR-BI null mice^3^. Among the spectrum of defective neurulation conditions conferred by abnormal closure at different portions of the neural tube, only cranial NTD is observed in SR-BI^−/−^ embryos.

During murine early development, SR-BI is not detected in the embryo itself but rather in trophoblast giant cells (TGC) from the parietal yolk sac^4, 5^. TGC play a critical role in embryonic uptake of various nutrients from the maternal blood supply before the establishment of a mature placenta^6^. Despite the prominent role of SR-BI in cholesterol uptake^1^ and the key role of cholesterol during embryonic development^7^, we have shown that the embryonic cholesterol content is similar in SR-BI^+/+^ and SR-BI^−/−^ embryos^5^.

In addition to cholesterol, HDL particles also transport other lipids, such as triglycerides, lipophilic vitamins and hydrophobic signalling molecules. Studies using adult SR-BI^−/−^ mice demonstrated lower levels of vitamin E in several SR-BI-expressing tissues, suggesting that beyond its role as a cholesterol transporter, this receptor also mediates the cellular uptake of vitamin E from HDL^8^. Vitamin E is a generic name for a group of isomers of two related molecules: tocopherols and tocotrienols. This vitamin was first described almost 100 years ago to be an essential factor for the success of pregnancy in rats^9^. Vitamin E isomers have antioxidant activity and intercalate between lipids in biological membranes, where they stop reactive oxygen species (ROS)-based reactions that generate lipoperoxides^10^. In addition, some isomers have other less well-defined biological activities, such as modulation of intracellular signalling pathways, gene expression and cell proliferation^11^. Vitamin E deficiency in rodents has been associated with congenital malformations, including neural tube defects (NTD)^12^. Consistent with this finding, this vitamin is effective in preventing NTD in mouse models of maternal diabetes-induced malformations, which are associated with an increased oxidative status^13^.

Considering this evidence, we hypothesized that NTD in SR-BI^−/−^ embryos was due to oxidative stress resulting from impaired vitamin E uptake from maternal circulation. To test this hypothesis, we studied the incidence of NTD, vitamin E content, ROS levels and gene expression in embryos and in TGC retrieved from dams fed with control or α-tocopherol-enriched diets.

## Results

### Effect of maternal α-tocopherol supplementation on NTD in SR-BI^−/−^ embryos

As a first approach to determine if defective maternal-embryonic vitamin E metabolism contributes to neural malformation in SR-BI^−/−^ embryos, we sought to analyse whether α-tocopherol supplementation in pregnant SR-BI^+/−^ dams reduced the incidence of NTD. The rationale behind this hypothesis was that if SR-BI is involved in vitamin E transport across the maternal foetal interface, then increasing the concentration of this vitamin in the maternal plasma may compensate for the lack of SR-BI via SR-BI-independent transport pathways. This possibility was strongly supported by our analysis of transcriptomic data in parietal TGC generated by Hannibal *et al*.^14^ which showed that, besides SR-BI, TGC express other proteins that are involved in lipoprotein metabolism, such as the HDL receptor Gpihbp1, receptors for others lipoproteins (e.g., Ldlr, Apobr, and Lrp1), and the lipid transporters Abca1 and Abcg1, which mediate lipid efflux from cells to Apoa1 and HDL, respectively (Table 1).

**Table 1:** Gene expression analysis^*#*^ of lipoprotein receptors and lipid transporters in trophoplast giant cells.

			Read count	
Group	Gene	Alternative name	mean	SD
Positive Controls	Prl3d1	Pl-1	170429	66683
	Prl3b1	Pl-2	495	196
	Prl2c2	Plf	466	219
Negative Controls	Tpbpa		32	13
	Ctsq		3	2
HDL Receptors	Scarb1	SR-BI	20331	7680
	Atp5b		1578	617
	Gpihbp1		444	261
LDL Receptors	Ldlr		1416	538
	Apobr		574	190
	Olr1	LOX-1	555	239
	Lrp10		551	208
Chilomicrons Receptors	Lrp1		187	84
Lipid Transporters	Abca1		142	61
	Abcg1		124	48

^#^Analysis of data generated by Hannibal, *et al*.^14^.

**Figure 1:**
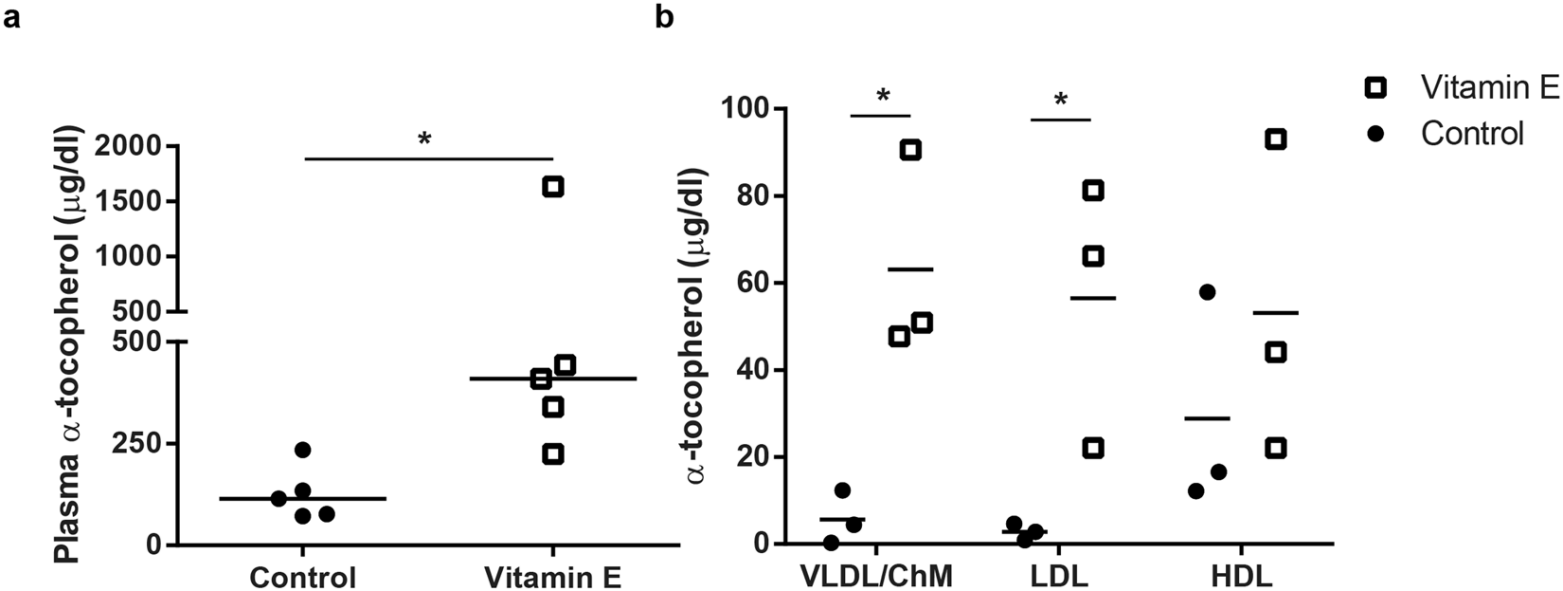
Plasmatic total (**a**) and lipoprotein (**b**) α-tocopherol levels in SR-BI^+/−^ pregnant dams fed with control or vitamin E supplemented diets. Concentrations of α-tocopherol in total plasma (**a**) or in different lipoproteins (**b**). VLDL/ChM: fraction containing very low density lipoproteins and chylomicrons. LDL: low density lipoproteins. HDL: high density lipoproteins. The horizontal lines indicate median (**a**) and mean (**b**). *p < 0.05, assessed by Mann-Whitney (**a**) or two-way ANOVA with Holms-Sidak post-test (**b**).

Because vitamin E supplementation has been shown to reduce plasma cholesterol levels in rodent models of hypercholesterolemia^15–17^ and because cholesterol has been shown to be crucial for embryonic development^7^, we analysed the impact of α-tocopherol supplementation on the plasma cholesterol levels of pregnant dams. Consumption of the vitamin-enriched diet did not affect the total circulating cholesterol levels nor its distribution into lipoproteins, as both parameters were similar in dams fed control or supplemented diets (Supplementary Fig. 1a and b). Alpha-tocopherol dietary supplementation from conception until gestational day 9.5 (E9.5) resulted in a significant rise in this lipid in maternal plasma (Fig. 1a). Interestingly, the vitamin E distribution in lipoproteins was affected by α-tocopherol supplementation. Compared to plasma from control mice, in which almost no α-tocopherol was detected in larger lipoproteins such as LDL, VLDL or chylomicrons, plasma from dams fed the vitamin E-enriched diet exhibited similar levels of α-tocopherol in HDL and non-HDL particles (Fig. 1b). In sum, the expression of other lipoprotein receptors than SR-BI in TGC (Table 1) and the detection of α-tocopherol in non-HDL lipoproteins in SR-BI^+/−^ dams fed the vitamin E-supplemented diet (Fig. 1b) suggest the co-existence of different α-tocopherol transport mechanisms from maternal blood into TGC, involving both HDL and non-HDL lipoproteins.

To analyse the impact of α-tocopherol supplementation on the incidence of NTD, embryos at E9.5 were retrieved from chow- or vitamin E-fed heterozygous dams and phenotypically classified as normal or as having NTD. Consistent with previous results from our group, a significant proportion of SR-BI^−/−^ embryos (54%) and a few SR-BI^−/+^ embryos (6%) from chow-fed SR-BI^−/+^ dams exhibited cranial NTD (Table 2). By contrast, only 1 out of 59 SR-BI^−/−^ embryos and 2 out of 129 SR-BI^−/+^ embryos (less than 2% in both cases) showed cranial NTD in vitamin E-supplemented SR-BI^−/+^ pregnancies (Table 2). Interestingly, α-tocopherol supplementation after implantation, starting from E4.5, also reduced the proportion of SR-BI^−/−^ and SR-BI^−/+^ embryos with NTD to levels comparable with supplementation from E0.5 to E9.5 (Table 3). Injecting pregnant dams with folic acid, a water-soluble vitamin that is well known to prevent NTD, reduced the percentage of SR-BI^−/−^ embryos with NTD from 56% to 19%, showing that NTD is folate-sensitive in SR-BI^−/−^ embryos (Table 4). The mean number of viable embryos that showed cardiac activity and the mean number of resorptions retrieved per dam were similar in the control, vitamin E- and folate-supplemented groups, indicating that embryonic viability and female fertility were not affected by these maternal interventions (Supplementary Table 2). As in our previous work, only cranial NTD, and not defective closure in the dorsal or posterior neural tube, was observed in SR-BI^−/−^ and SR-BI^−/+^ embryos, irrespective of treatment. In summary, α-tocopherol dietary supplementation, starting either after mating or at implantation, proved to be a highly efficient intervention to prevent NTD in SR-BI-deficient embryos.

**Table 2:** Incidence of NTD in SR-BI^+/−^ and SR-BI^−/−^ embryos obtained from SR-BI^+/−^ dams fed chow or vitamin E-supplemented diets from E0.5 to E9.5.

Treatment	SR-BI^+/+^ no. (%)			SR-BI^+/−^ no. (%)			SR-BI^−/−^ no. (%)		
	Normal	NTD	Total	Normal	NTD	Total	Normal	NTD	Total
Control	70 (100%)	0 (0%)	70	163 (94%)	10 (6%)	173	34 (46%)	40 (54%)	74
Vitamin E	49 (100%)	0 (0%)	49	127 (98%)	2 (2%)	129	58 (98%)	1 (2%)^a^	59

^a^p < 0.001 vs. Control; Fisher’s exact test.

**Table 3:** Incidence of NTD in SR-BI^−/−^ embryos from SR-BI^+/−^ dams fed chow or vitamin E-supplemented diets starting from conception (E0.5) or from implantation (E4.5) until E9.5.

Treatment	SR-BI^+/+^ no. (%)			SR-BI^+/−^ no. (%)			SR-BI^−/−^ n° (%)		
	Normal	NTD	Total	Normal	NTD	Total	Normal	NTD	Total
Control	9 (90%)	1 (10%)	10	26 (90%)	3 (10%)	29	7 (50%)	7 (50%)	14
Vitamin E	21 (100%)	0 (0%)	21	33 (100%)	0 (0%)	33	12 (92%)	1 (8%)^a^	13
Vitamin E 4–9	15 (94%)	1 (6%)	16	26 (96%)	1 (4%)	27	13 (93%)	1 (7%)^a^	14

^a^p < 0.05 vs. Control; Fisher’s exact test.

**Table 4:** Incidence of NTD in SR-BI^−/−^ embryos from SR-BI^+/−^ dams injected with folic acid or vehicle.

Treatment	SR-BI^+/+^ no. (%)			SR-BI^+/−^ no. (%)			SR-BI^−/−^ no. (%)		
	Normal	NTD	Total	Normal	NTD	Total	Normal	NTD	Total
Control	27 (100%)	0 (0%)	27	58 (97%)	2 (3%)	60	12 (44%)	15 (56%)	27
Folate	29 (94%)	2 (6%)	31	85 (96%)	4 (4%)	89	30 (81%)	7 (19%)^a^	37

^a^p = 0.05 vs. Control; Fisher’s exact test.

### Vitamin E content in embryos and parietal yolk sacs lacking SR-BI

**Figure 2:**
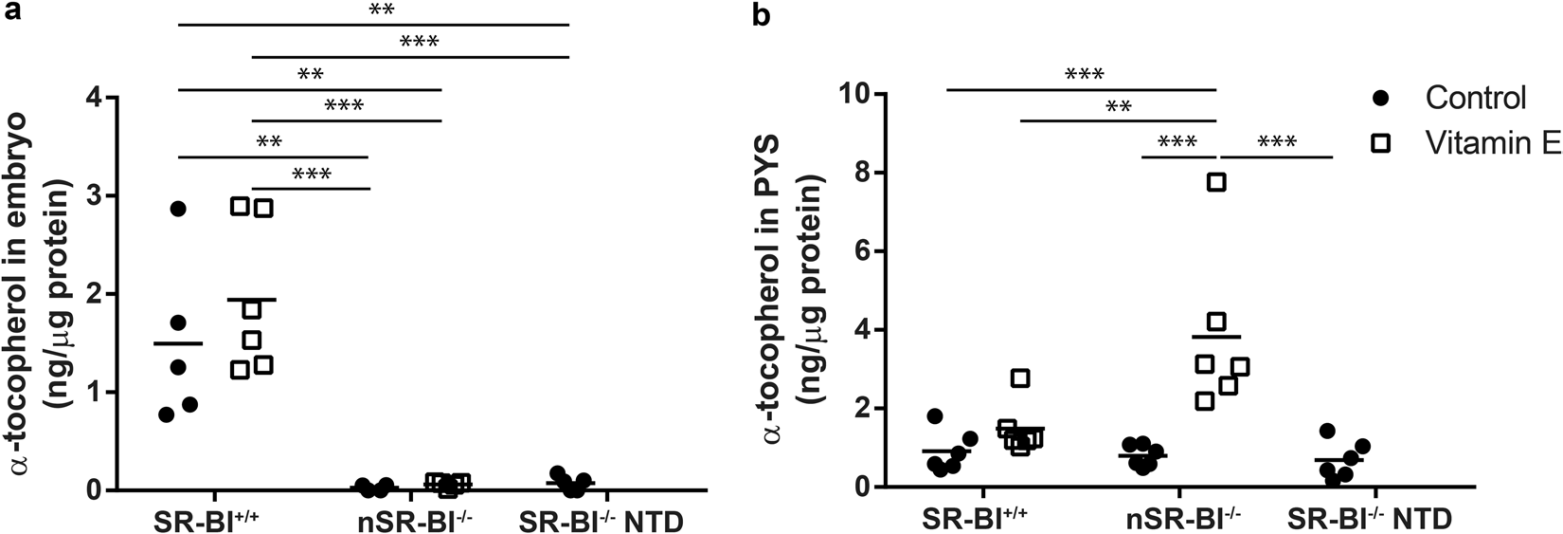
Vitamin E content in embryos (**a**) and parietal yolk sac (PYS) (**b**) obtained from SR-BI^+/−^ dams fed with control or vitamin E supplemented diets. Levels of α-tocopherol were measured in pairs of wild-type embryos (SR-BI^+/+^), normal knock-out embryos (nSR-BI^−/−^) and knock-out embryos with NTD (SR-BI^−/−^ NTD) (**a**) and in single PYS (**b**). **p ≤ 0.01, ***p ≤ 0.001, determined by one-way ANOVA and Tukey’s post-test.

We next sought to determine whether the lack of SR-BI affected the vitamin E content in SR-BI^−/−^ mouse embryos and TGC, as previously reported for certain adult tissues^8^. Supporting the notion that SR-BI is involved in vitamin E maternal-embryonic provision, α-tocopherol quantification in SR-BI^−/−^ embryos showed a 50-fold reduction compared to SR-BI^+/+^ embryos (Fig. 2a). Two unexpected results were obtained from these experiments: i) the very low vitamin E content was similar in SR-BI^−/−^ embryos with NTD (SR-BI^−/−^ NTD) or with normal neural tube closure (nSR-BI^−/−^) and ii) maternal supplementation with α-tocopherol did not increase the embryonic vitamin E levels, despite its striking effect on NTD prevention (Fig. 2a).

SR-BI is specifically expressed in TGC, but these large cells are tightly adhered to small parietal endoderm cells by a thick extracellular matrix constituting the parietal yolk sac (PYS). In order to avoid potential modifications in the metabolic or transcriptional status of TGCs due to isolation procedures, we determined the content of α-tocopherol in the PYS as a whole. The α-tocopherol content was similar in PYS from SR-BI^+/+^ and SR-BI^−/−^ embryos with or without NTD obtained from chow-fed dams (Fig. 2b). Although maternal supplementation with α-tocopherol did not increase the vitamin E content in SR-BI^+/+^ PYS, the vitamin E content was almost 5-fold higher in SR-BI^−/−^ PYS.

### Redox status in SR-BI^−/−^ embryos and TGC

**Figure 3:**
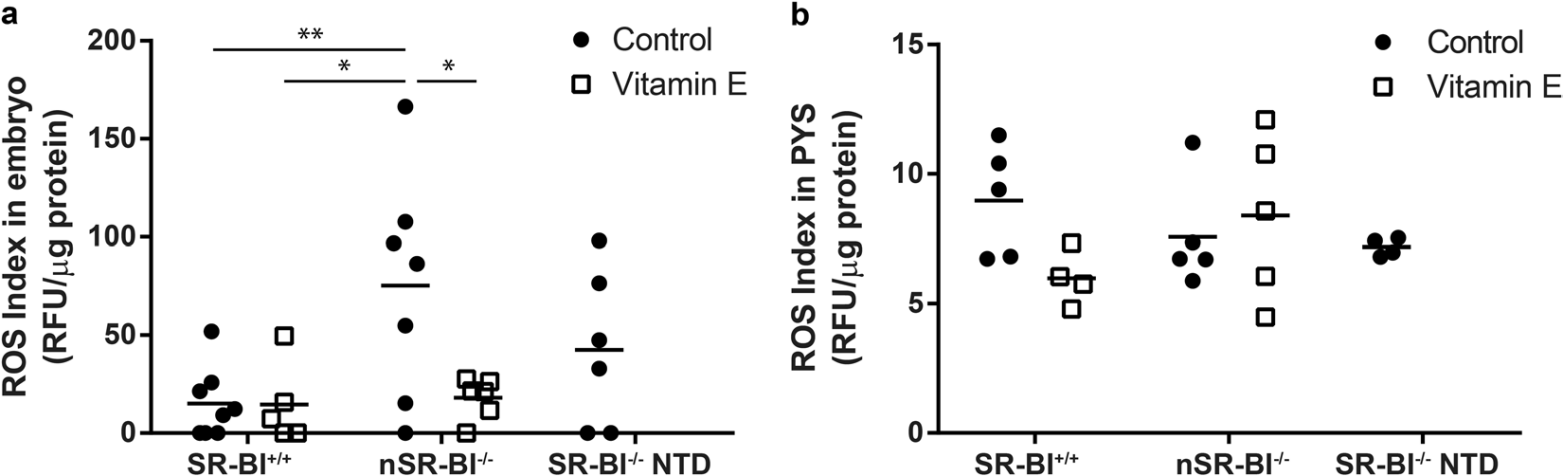
ROS index in embryos (**a**) and parietal yolk sac (PYS) (**b**) obtained from SR-BI^+/−^ dams fed with control or vitamin E supplemented diets. Levels of reactive oxygen species were measured using DCF-DA in pairs of wild-type embryos (SR-BI^+/+^), normal knock-out embryos (nSR-BI^−/−^) and knock-out embryos with NTD (SR-BI^−/−^ NTD) (**a**) and single PYS (**b**). *p ≤ 0.05, **p ≤ 0.01, determined by one-way ANOVA and Tukey’s post-test.

Because accumulation of oxidative species has been shown to impair neural tube closure in mice^13^, we determined the reactive oxygen species (ROS) levels by fluorimetry in vitamin E-deficient SR-BI^−/−^ embryos (see methods and Supplementary Fig. 2). In chow-fed dams, nSR-BI^−/−^ embryos exhibited a 5-fold higher fluorescence intensity compared to SR-BI^+/+^ embryos (Fig. 3a). Surprisingly, NTD SR-BI^−/−^ embryos showed intermediate fluorescence levels compared to SR-BI^+/+^ and nSR-BI^−/−^. Maternal α-tocopherol supplementation resulted in significantly lower ROS levels in SR-BI^−/−^ embryos, similar to the fluorescence levels in SR-BI^+/+^ embryos. Trophoblast giant cells have been shown to produce significant ROS due to high activity of the enzyme NADPH oxidase^18^. However, SR-BI deficiency did not affect ROS levels in parietal yolk sacs from embryos obtained from mothers fed control or vitamin E-enriched diets (Fig. 3b). Together, these results show that excess ROS in SR-BI^−/−^ embryos can be prevented by maternal α-tocopherol supplementation.

**Figure 4:**
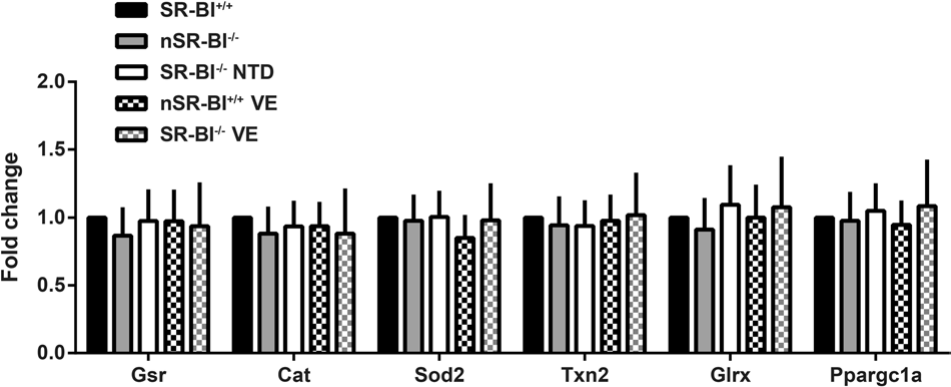
Antioxidant gene expression response in embryos obtained from SR-BI^+/−^ dams fed with control or vitamin E supplemented diets. Expression levels of genes activated during antioxidant response were measured in pools of 3 wild-type embryos (SR-BI^+/+^), normal knock-out embryos (nSR-BI^−/−^) and knock-out embryos with NTD (SR-BI^−/−^ NTD). N = 3 pools per group.

High ROS production and oxidative stress are associated with an antioxidant response that is characterized by up-regulation of the expression of several enzymes that metabolize ROS into harmless compounds. Using real time PCR, we compared the expression of genes encoding antioxidant enzymes in SR-BI^+/+^ and SR-BI^−/−^ embryos (with or without NTD) as well as in their respective PYS, from dams fed chow or vitamin E-enriched diets. Our results showed similar expression of *Gsr*, *Cat*, *Sod2*, *Txn2* and *Glrx* among embryos of different genotypes, phenotypes or maternal diets (Fig. 4). We also evaluated the expression of *Ppargc1a*, a target and master regulator of the antioxidant response^19^, and found that its expression was also similar among the embryos from different groups (Fig. 4). In PYS, minor changes with unclear biological significance were found in *Cat*, *Txn2* and *Glrx* gene expression (Supplementary Fig. 3), independent of genotype, phenotype, or dietary treatment. We did not compare the expression of *Ppargc1a* in PYS because the mRNA levels were below the detection limit in this tissue.

### Expression of genes involved in neural tube closure in SR-BI^−/−^ embryos

**Figure 5:**
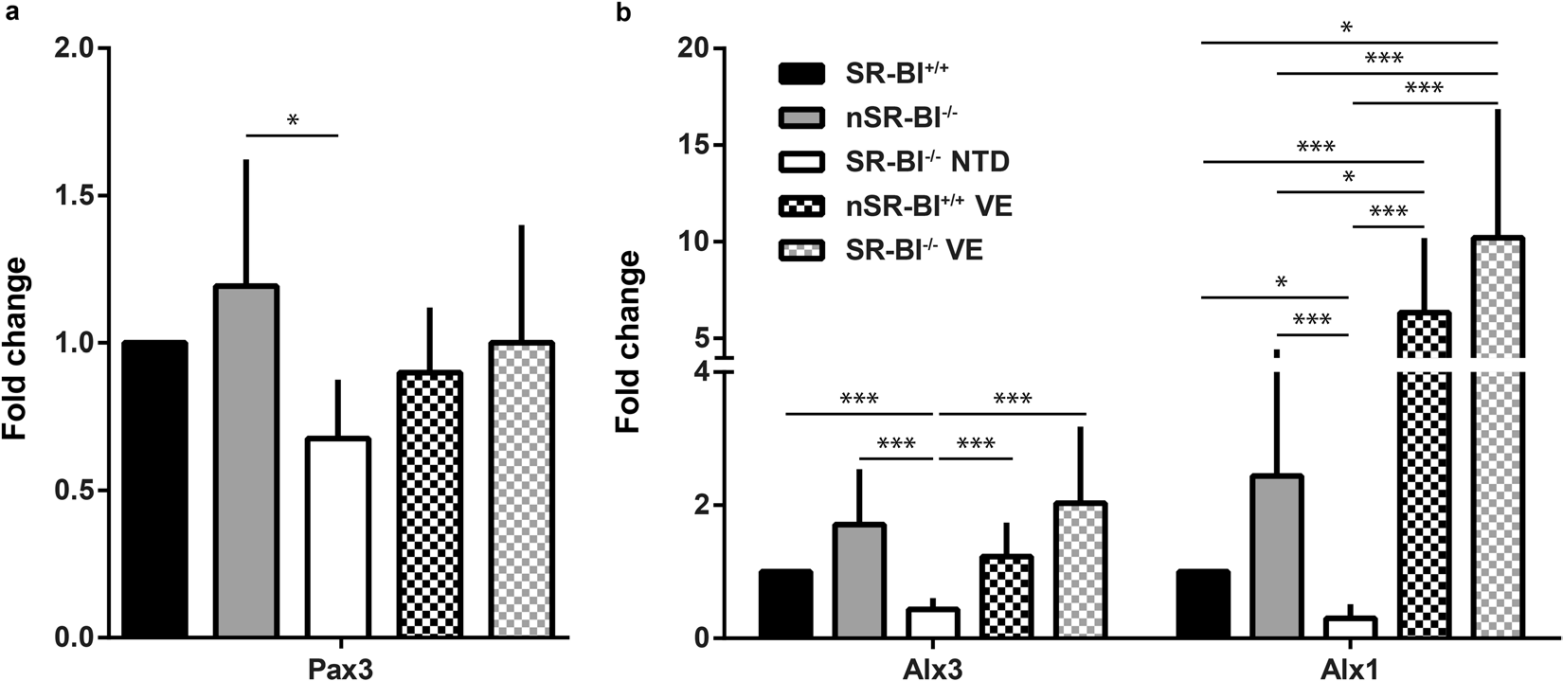
Expression of neural tube closure-related transcription factors in embryos obtained from SR-BI^+/−^ dams fed with control or vitamin E supplemented diets. Expression levels of Pax3 (**a**) and Alx transcription factors (**b**) were determined in pools of 3 E9.5 wild-type embryos (SR-BI^+/+^), normal knock-out embryos (nSR-BI^−/−^) and knock-out embryos with NTD (SR-BI^−/−^ NTD). N = 3 pools per group.

As gene expression orchestrates the molecular and cellular processes that take place during neural tube closure, many mutations in mouse genes coding for transcription factors give rise to NTD^20^. Thus, we tested whether abnormal gene expression contributed to NTD in SR-BI^−/−^ embryos and whether this expression was normalized after maternal α-tocopherol supplementation. We compared the mRNA levels for genes known to be relevant for neural tube closure in SR-BI^+/+^ and SR-BI^−/−^ embryos of both phenotypes (normal or NTD) obtained from control chow- or vitamin E-fed dams. We first analysed the expression of genes coding for proteins involved in the Hedgehog (Hh) signalling pathway, one of the main regulators of neural tube closure and neuronal specification^21, 22^. We observed similar expression for Hh gene targets in SR-BI^−/−^ embryos compared to SR-BI^+/+^ embryos (Supplementary Fig. 4). We also checked the mRNA levels for *Pax3*, a key paired-box transcription factor whose inactivation leads to NTD with total penetrance in the Splotch mouse^23, 24^. *Pax3* expression is significantly reduced in murine models of maternal diabetes, in association with an embryonic accumulation of ROS and a partially penetrant NTD phenotype^13^. Our results showed altered *Pax3* expression, specifically in NTD SR-BI^−/−^ embryos from chow-fed dams compared to nSR-BI^−/−^ (Fig. 5a). We also examined gene expression of two members of the aristaless-like (Alx) homeobox protein family that are involved in neural tube closure. One of these genes is *Alx3*, whose inactivation induces a partially penetrant NTD phenotype^25^. Interestingly, reduction in *Alx3* expression is observed in mouse embryos deficient for Lrp2, a multiligand receptor mediating HDL endocytosis^26^. Our results showed that expression of *Alx3* was significantly reduced in NTD SR-BI^−/−^ embryos compared to SR-BI^+/+^ embryos and to nSR-BI^−/−^ embryos (Fig. 5b). Maternal treatment with vitamin E normalized *Alx3* expression in SR-BI^−/−^ embryos. Another member of the aristaless-like family of proteins that is involved in neural tube closure is *Alx1* ^27^, which has an expression domain and function that are partly redundant with *Alx3* ^28^. *Alx1* expression in NTD SR-BI^−/−^ embryos was 8-fold lower than that in nSR-BI^−/−^ embryos (Fig. 5b). Regardless of genotype, embryos from vitamin E-supplemented dams had higher *Alx1* expression than embryos from chow-fed dams.

## Discussion

Although several lipids have been shown to be crucial for early development, the molecular mechanisms explaining the transport of these molecules between the mother and embryo or foetus are still not completely understood. Early embryonic nutrition is accomplished by the transport of nutrients from maternal endometrial glands to the embryo through TGC and visceral endoderm cells of the yolk sac. Given the expression of SR-BI in TGC and the high incidence of NTD in SR-BI^−/−^ mouse embryos^4, 5^, we assessed the role of SR-BI in embryonic vitamin E uptake and its implications for neural tube closure. Our main findings are that SR-BI^−/−^ embryos exhibit defective embryonic vitamin E levels and that maternal α-tocopherol supplementation can almost completely prevent NTD in SR-BI^−/−^ embryos.

In rodents, vitamin E is essential for the success of pregnancy, and maternal deficiency of this vitamin has been associated with NTD in offspring, among other malformations^9, 29^. In one study describing embryos lacking the main intracellular α-tocopherol binding protein (Ttpa), researchers suggested that vitamin E is required for placental formation and not essential for embryo development itself^30^. Our results support the idea that uptake of this nutrient is required for neural tube closure during early development, even before the establishment of a fully functional placenta.

Impaired lipoprotein metabolism in the maternal-embryonic interface has been previously linked to neural tube closure abnormalities. For example, genetic inactivation of microsomal triglyceride transfer protein (Mttp) impairs lipoprotein packaging in the endoplasmic reticulum of the visceral endoderm and is associated with embryonic lethality and cranial NTD^31^. Apob-containing lipoproteins have been shown to be secreted from the visceral endoderm into the embryonic environment^32^. In various mouse models with inactivating mutations in the Apob gene, homozygosity leads to embryonic lethality, characterized by the presence of cranial NTD in a subset of the mutants^33–35^. Interestingly, embryos lacking Apob have slightly lower cholesterol than wild-type embryos and extremely low levels of vitamin E^36^, providing further support to the idea that reduced transport of this vitamin may impair neural tube closure. However, in contrast to SR-BI^−/−^ embryos, maternal vitamin E supplementation of dams that are heterozygous for the Apob mutation does not prevent NTD in homozygous embryos^37^. It is possible that the visceral endoderm entirely relies on Apob and Mttp to secrete vitamin E to Apob-containing lipoproteins, rendering maternal supplementation ineffective. By contrast, TGC may rely on other vitamin E transport mechanism besides SR-BI, as suggested by the expression of diverse proteins that are involved in lipoprotein uptake by receptor-mediated endocytosis in extraembryonic tissues. These pathways might be efficient enough to compensate for the lack of SR-BI in SR-BI^−/−^ TGC, providing them with vitamin E after maternal overloading of this nutrient. The redistribution of this vitamin into different classes of lipoproteins in α-tocopherol-supplemented dams further supports this possibility.

An unexpected result was that, despite the high levels of vitamin E in SR-BI^−/−^ TGC and prevention of NTD in SR-BI^−/−^ embryos, embryonic vitamin E levels were not restored by maternal supplementation with α-tocopherol. Among the possibilities that could explain this finding is that vitamin E transported by SR-BI-independent mechanisms may be oxidized or metabolized rapidly before or during transport to the embryo, becoming undetectable by the methods used. Future experiments involving the detection of vitamin E metabolites in PYS and embryos could aid in testing this hypothesis. Alternatively, there may be an indirect effect on the embryo caused by vitamin E loading of TGC. Although this possibility is not supported by our results, which show similar levels of ROS and mRNA for genes from the antioxidant response in PYS of different genotypes or phenotypes, the use of whole PYS instead of TGCs might have reduced the sensitivity of the assay. In this work, TGCs were not separated from parietal endoderm cells to avoid the potential effects of the isolation procedure on the cellular composition.

Among various genetic and environmental causes of NTD identified in both human and animal experimental studies, nutrient inadequacies are important factors increasing the susceptibility to defective neural tube closure. Over the last few decades, maternal periconceptional supplementation with folic acid has proven to be a safe and efficient intervention to reduce the incidence of human and experimental NTD. However, both folate responsive and folate-resistant NTD have been detected in human and animal embryos, leading researchers to propose the use of combined therapies including folate and other nutrients, such as inositol^38^ or multivitamin supplements^39^, to reduce NTD. In this work, our results show that cephalic defective neurulation in SR-BI-deficient embryos can be reduced by supplementing dams with folate or vitamin E. Although we cannot compare the effectiveness of each intervention due to the use of unique doses and different administration routes, our findings of vitamin E deficiency and NTD prevention in SR-BI null embryos support the idea that other nutrients besides folate need to be considered for the prevention of NTD, given the complex and heterogeneous aetiology of this condition.

One of the principles underlying NTD and other congenital malformations in rodents is excessive embryonic oxidative stress, which is observed *in vivo* in rodent models of maternal diabetes^13^ and ethanol consumption^40^, and in mice deficient for thioredoxin 2 (Txn2), a protein scavenging ROS in mitochondria^41^. Vitamin E has proven to be effective in preventing ROS-induced NTD in murine models both *in vivo* ^13, 42^ and *in vitro* ^43^. In this work, normalization of ROS levels in SR-BI^−/−^ embryos after maternal α-tocopherol supplementation suggests an antioxidant effect of this vitamin E^13^. However, we cannot rule out, at present, the existence of additional non-antioxidant effects of α-tocopherol contributing to the results described.

It is worth noting that although all SR-BI^−/−^ embryos had low levels of vitamin E, only approximately half of the embryos exhibited NTD. The incomplete penetrance of NTD in isogenic embryos that developed in a homogeneous uterine environment has been previously observed in mouse models^20^. Phenotypic discordance for disease susceptibility has also been shown in monozygotic human twins^44, 45^, including twins discordant for anencephaly^46^. Studies in *C*. *elegans* have suggested that inter-individual stochastic variations in gene expression and activation of compensatory mechanisms could account for different phenotypic consequences of mutations in those organisms^47^. Similarly, we detected that SR-BI^−/−^ embryos that underwent neural tube closure exhibited higher mRNA levels for a subset of genes involved in neural tube closure compared to SR-BI^−/−^ with NTD, including Pax3 and 2 genes of the aristaless-like family (Alx1 and Alx3). The fact that SR-BI^−/−^ embryos obtained from vitamin E-supplemented dams exhibited similar or even higher expression of those genes than embryos from chow-fed dams suggests that normalization of the expression of those genes might contribute to the prevention of NTD in our model. It cannot be discounted, however, that deficiencies in mRNA levels for those genes may be a consequence and not a cause of failed neurulation.

Despite the involvement of SR-BI in human cholesterol homeostasis and cardiovascular function, no null mutations and very few loss-of-function mutations in the human gene coding for SR-BI have been reported^48, 49^. Considering that SR-BI mutations are very rare in humans and that SR-BI is expressed in first trimester human trophoblasts, it could be speculated that null or severely deficient SR-BI gene expression might hinder human embryonic development. In humans, vitamin E supplementation during mid and late pregnancy has proven to be inefficient in preventing stillbirth, neonatal death, preterm birth, pre-eclampsia or poor foetal growth^50^. Two retrospective studies have suggested a relationship between maternal vitamin E consumption, estimated through food questionnaires, and offspring NTD^51, 52^. In one of the studies, a reduction of the incidence of anencephaly, and not of other NTD, was only observed in the third quartile of vitamin E consumption^51^. In the second study, a higher intake of vitamin E and other micronutrients was associated with decreased risk of spina bifida^52^. Although the incidence of NTD in various human populations has been successfully reduced by folic acid consumption, in particular in countries where primary preventive strategies have been implemented^53^, this malformation has not completely been abolished. In this regard, ensuring adequate vitamin E levels by appropriate nutritional counselling for pregnant women could aid in reducing the remaining risk of human NTD. This could be especially beneficial for pregnant women with disorders associated with a higher oxidative status, such as obesity and type 1 and 2 diabetes, all of which exhibit a higher risk for NTD^54–56^. Short-term vitamin E supplementation, although not free from potential associated risks, could also be envisioned as a potential intervention for women willing to conceive after one or more previously folate-resistant NTD-affected pregnancies.

## Methods

### RNA-Seq analysis pipeline

We analysed the dataset generated by Hannibal *et al*.^14^, which was produced by RNA sequencing of polyA^+^ RNA from isolated mouse E9.5 parietal TGC. The Single End Reads RNA raw sequence files (.fastq files) were extracted from GEO Accession Number GSE50585. We used the entries GSM1223565, GSM1223566, GSM1223567 and GSM1223568 to generate our data, which underwent quality control analysis using FastQC (http://www.bioinformatics.babraham.ac.uk/projects/fastqc/). Single End RNAseq reads were groomed using FASTQ groomer V1.04^57^, and groomed reads were mapped to the mouse genome (mm10) using TopHat2 version 0.7^58^. Aligned reads were counted using HTseq-count Version 1.0.0^59^ to generate a digital expression matrix. To ensure that all of the sequences were processed consistently, all of the above steps were performed as part of a Galaxy workflow, which can be found at https://usegalaxy.org/u/laiumiunix/w/rnaseq.

A list of all of the lipoprotein receptors was obtained from the Gene Ontology Consortium using the “Lipoprotein particle receptor activity” and “Regulation of plasma lipoprotein particle levels” search terms. The positive controls were marker genes of parietal TGC, whereas the negative controls were markers of TGC from a different lineage that does not give rise to parietal TGC^60^. We considered a gene to be expressed by TGC when the mean read count of that gene was superior to the mean +3 SD of Tpbpa, the negative control with the highest count. This criterion set the threshold at 71 mean reads.

### Animals

We used mice in a mixed C57Bl6/J ×129 background carrying a targeted mutation in the SR-BI locus, generously provided by Monty Krieger (B6;129S2-Scarb1^tm1Kri^/J)^3^. Animals were maintained in the animal facility of the Department of Gastroenterology (School of Medicine, Pontificia Universidad Católica de Chile) at 25 °C and 12 h light:dark cycling and fed standard chow (Prolab RMH3000, Labdiet; 75 IU vitamin E/kg) and water *ad libitum*. Protocols were conducted in agreement with the National Research Council (NRC) publication Guide for Care and Use of Laboratory Animals (copyright 1996, National Academy of Science). Studies were approved by the Ethics Committee for Animal Welfare from the School of Medicine of the Pontificia Universidad Católica de Chile.

To generate intercrosses, 2- to 4-month-old heterozygous (SR-BI^+/−^) females were caged with 2- to 6-month-old SR-BI^+/−^ males at a 1:1 or 2:1 ratio. Female mice were checked daily for the presence of a vaginal plug during the first hour of the light cycle. The day a plug was detected was recorded as embryonic day 0.5 (E0.5) and the female was separated from the male and maintained with one or two females in the same cage. All embryos were collected on day E9.5, when neural tube closure is complete in wild-type embryos. Pregnant dams were anaesthetized with a mixture of ketamine:xylazine (0.18 mg:0.012 mg per gram of body weight) and the peritoneal cavity was exposed. A blood sample was taken from the abdominal vena cava and uteri were excised. Implantation sites were retrieved and the embryos and PYS were collected. Embryos were assessed for neural tube closure and classified into normal or NTD. The visceral yolk sac was snap frozen in liquid nitrogen, preserved at −80 °C and used for individual genotyping. Embryo and PYS samples were also immediately frozen in liquid nitrogen and stored individually at −80 °C until use. As each dam produced variable numbers of embryos with different genotypes and phenotypes, pools of embryos retrieved from different dams were used.

For all experiments analysing the impact of vitamin E dietary supplementation, pregnant dams were fed α-tocopherol-enriched chow (Prolab 5P00 diet enriched with 2,000 I.U. α-tocopherol/kg, PMI Nutrition International, Richmond, IN) only after mating, from E0.5 to E9.5. Only in the experiment shown in Table 3, some of the dams were fed with the vitamin E-enriched diet from E4.5 to E9.5. The diet was kept at −20 °C until use and stored for no longer than 18 months, and feed in the cages was changed at least every two days. The α-tocopherol-enriched diet contained 1.1 ppm folic acid, which was very similar to the folic acid concentration in the chow diet.

Folic acid (Sigma, MO) in PBS was injected i.p. daily (10 mg/kg body weight)^61^ to pregnant dams from E0.5 to E9.5. As a control, pregnant dams were injected i.p. with PBS during the same period.

### Lipoprotein separation

Whole plasma (200 µl) was chromatographically fractionated by Fast Protein Liquid Chromatography (FPLC) using a Superose-6 column (GE Life Sciences, PA). The mobile phase was an aqueous solution at pH 7.4 containing 150 mM NaCl and 1 mM EDTA, at a constant flow of 9 ml/hour. The first 15 ml was discarded, and then 40 fractions of 300 µl each were recovered. To determine the α-tocopherol content of the various lipoprotein classes, the fractions corresponding to each lipoprotein class (as indicated in Supplementary Fig. 1B) were pooled.

### Cholesterol determination

Cholesterol in whole plasma (10 µl) and FPLC fractions (300 µl) was measured with the enzymatic method reported by Allain *et al*.^62^. Samples were incubated for 30 minutes at 37 °C with reaction buffer (0.5 M Tris pH 7.6, 50 mM phenol, 50 mM 4-chlorophenol, 1% Triton X-100, 0.37% sodium cholate, 0.04% 4-aminoantipyrine, 0.35 U/ml cholesterol esterase, 0.1 U/ml cholesterol oxidase, 1.1 U/ml peroxidase). The absorbance was measured at 490 nm in a plate reader.

### Vitamin E determination

Pairs of embryos or single PYS were lysed in RIPA buffer containing 1% N-acetylcysteine and centrifuged at 12,000 g at 4 °C for 10 minutes. Lysates, as well as whole plasma (200 µl) and pooled FPLC fractions (1.5 to 3 ml), were extracted for 1 hour in 500 µl methanol (0.01% butylated hydroxytoluene) and 5 ml hexane. Methanol and hexane mixtures were centrifuged for 5 minutes at 1,000 g at room temperature, and the supernatants were recovered and dried under nitrogen. Dried extracts were resuspended in methanol:ethanol 1:1 (200 µl of mixture) and filtered through a 0.22-µm PTFE filter. The samples were subjected to HPLC through a Symmetry LC-8 reverse phase column (Waters, MA). The mobile phase used was 20 mM sodium perchlorate in methanol:water 97.5:2.5 at a constant flux of 1 ml/minute controlled by a L-6000 HPLC Pump (Hitachi, IL). Elution signal was detected with a LC-4C amperometric detector (Bioanalytical Systems, Inc., IN) at 600 mV vs. Ag/AgCl. The samples were run in triplicate and the signal was interpolated in a standard curve for α-tocopherol. Among the various vitamin E isomers, the only detectable isomer in both PYS and embryos was α-tocopherol.

### Reactive oxygen species analysis

The presence of reactive oxygen species was detected using the method from Chen^63^ with modifications. Pairs of embryos and single parietal yolk sacs were kept on ice and lysed by sonication for 2 seconds in 100 µl reaction buffer (130 mM KCl, 5 mM MgCl_2_, 20 mM KH_2_PO_4_, 20 mM Tris, 30 mM glucose). The lysates were incubated with dihydrodichlorofluorescein diacetate (DCF-DA; Sigma, MO) at a final concentration of 50 µM, and fluorescence was detected continuously for 1 hour at 37 °C with an excitation wavelength of 485 nm and a detection wavelength of 535 nm in a plate reader. For each sample, fluorescence was adjusted based on the protein content. Representative results of this assay for embryos and PYS are shown in Supplementary Fig. 2. Negative controls, including lysate-free samples and DCF-DA free samples, showed no fluorescence. As a positive control, lysates were incubated with H_2_O_2_ (100 µM final concentration) alone or together with N-acetylcysteine (NAC; 0.1% final concentration) just prior to the addition of DCF-DA. Incubation of lysates with H_2_O_2_ resulted in a significant increase in fluorescence that was reduced by incubation of lysates with H_2_O_2_ and NAC.

### Sex determination

Individual sexing of embryos was performed by allele discrimination using PCR, as described previously^64^. The primer sequences F: 5′-CCGCTGCCAAATTCTTTGG-3′ and R: 5′-TGAAGCTTTTGGCTTTGAG-3′ were used to amplify the bands corresponding to the smcx and y alleles in the X and Y chromosomes, respectively.

### Real time PCR

Total RNA was extracted from pools of 3 female embryos and individual parietal yolk sacs using the PureLink RNA Micro Kit (Invitrogen, CA), following the manufacturer’s instructions. The embryos used to make the pools came from 9 control litters and 6 vitamin E-supplemented litters. RNA integrity was evaluated using the Bioanalyser 2100 (Agilent, CA) with the Eukaryote Total RNA Nano assay (Agilent, CA). All samples had an RNA Integrity Number of 10. Purified RNA (500 ng) was used for retrotranscription with the iScript RT Supermix (Biorad, CA). The resulting cDNA was amplified by real time PCR with a StepOnePlus thermocycler (Applied Biosystems, CA) using the PowerUp SYBR Green master mix (Thermo, MA) and 100 nM of each primer. The primers, annealing temperatures and amplification efficiencies are listed in Supplementary Table 1. All primers were designed using NCBI’s Primer-BLAST^65^. The amplification conditions were as follows: 5 minutes at 95 °C and 40 cycles of 15 seconds at 95 °C, 15 seconds of annealing and 30 seconds at 72 °C. After every reaction, a melting curve was performed to ensure the amplification of a single product. The amplification efficiency of each pair of primers was determined by serial dilution of a mixture of the cDNAs. Then, the relative expression was calculated for each sample using the equation by Pfaffl^66^ (equation 1 in the reference) and the TATA-box binding protein (Tbp) as reference gene.

### Statistics

Sample sizes were calculated to achieve an 80% power of detecting a 2-fold change with α = 0.05. For supplementation with α-tocopherol after implantation, we sought an 80% power to detect a 95% reduction in the presence of NTD in SR-BI^−/−^ embryos with α = 0.05.

The assignment of pregnant dams to each treatment group was pseudo-randomized. Each day, the first female with a vaginal plug was assigned to the control group, the second one to one of the treatment groups, and so on. If only one female had a plug one day, the next day the order was reversed. The phenotypic assessments were performed blinded to the genotype of the embryo, but not to the treatment group. Biochemical and real time PCR experiments were performed blind to the genotype and the treatment group of the sample.

Results are shown as scatter plots with a horizontal line indicating the mean (or median where indicated) for arithmetic data, mean ± SEM for lipoprotein profiles and geometric mean + error for exponential data obtained from real time PCR experiments. The error represents the uncertainty in estimating the relative expression and is computed using Taylor’s series relative to the control group^67^. Therefore, error is reported only for the non-control groups.

The statistical significance of the difference between proportions was evaluated with the Fisher’s exact test. Differences between arithmetical means were tested for significance using one-way ANOVA with a Tukey’s post-hoc test or two-way ANOVA with the Holms-Sidak post-test. If variances were different between groups, then a non-parametric test was used (Mann-Whitney for two group comparison and Kruskal-Wallis with Dunn’s post-test for multigroup comparison). The significance of the difference in gene expression was tested using the Pair-wise Fixed Reallocation Randomization test with the Relative Expression Software Tool^67^ Multiple Comparison Solution^14^.

All tests were two-sided, and results were considered significant at p ≤ 0.05. The statistically significant differences between groups are symbolized by asterisks (*p ≤ 0.05, **p ≤ 0.01, ***p ≤ 0.001).

## Electronic supplementary material

Supplementary Tables and Figures
