# Supplementary material for: Deficient Vitamin E Uptake During Development Impairs Neural Tube Closure in Mice Lacking Lipoprotein Receptor SR-BI
Source: Sci Rep. 2017 Jul 12;7:5182. doi: 10.1038/s41598-017-05422-w (PMC5507922; doi:10.1038/s41598-017-05422-w)
Supplement: Supplementary file 1 — Supplementary Tables and Figures [file 41598_2017_5422_MOESM1_ESM.doc]

**Deficient Vitamin E Uptake During Development Impairs Neural Tube Closure in Mice Lacking Lipoprotein Receptor SR-BI**

Nicolás Santander^1^, Carlos Lizama^3^, María José Parga^1^, Alonso Quiroz^1^, Druso Pérez^2^, Guadalupe Echeverría^2^, Lorena Ulloa^4^, Verónica Palma^4^, Attilio Rigotti^1,2^ and Dolores Busso^1,*^

^1^*Department of Nutrition, Diabetes and Metabolism and* ^2^*Center of Molecular Nutrition and Chronic Diseases, School of Medicine, Pontificia Universidad Católica de Chile, Santiago, Chile.*

^3^*Cardiovascular Research Institute, University of California, San Francisco, California, USA.*

^4^*Laboratory of Stem Cell and Developmental Biology, Faculty of Sciences, Universidad de Chile, Santiago, Chile.*

**Supplementary Tables**

**Supplementary Table 1. Characteristics of the primers used in real time** PCR experiments.

| **Gene** |  | **Primer** | **Annealing T°** | **Efficiency (Embryos)** | **Efficiency (PYS)** |  |
| --- | --- | --- | --- | --- | --- | --- |
| Tbp | F | CAGATGTGCGTCAGGCGTTC | 57°C | 1.93 | 2.02 |  |
|  | R | AGAAACCTAGCCAAACCGCC |  |  |  |  |
| Gsr | F | CAGCCGGAAAGAAAGCGATG | 57°C | 1.9 | 1.99 |  |
|  | R | ACGTGGAATCCGTACTAGCG |  |  |  |  |
| Cat | F | AAGTCACCACTCCAGCGGGC | 57°C | 1.91 | 2.15 |  |
|  | R | GCTGCGGAACGTGAGACAGCA |  |  |  |  |
| Sod2 | F | TCGCTGTGTCCTTGCGGACG | 57°C | 1.84 | 1.99 |  |
|  | R | ACACGACCGCTGCTCTCCTCA |  |  |  |  |
| Txn2 | F | TTCCTGCCCTGCCCCTCTGTTT | 57°C | 1.92 | 2.02 |  |
|  | R | AGGGCTGGCTCTGAAGTGCT |  |  |  |  |
| Glrx | F | GCCGCTGCATTACCGGACCA | 57°C | 2.03 | 1.96 |  |
|  | R | CTCCTGAGCCATGCTGACAGGC |  |  |  |  |
| Ppargc1a | F | AGTCGAAAACCCAGAAAGTGC | 57°C | 1.8 | ND |  |
|  | R | GTCTTCGGGATTGTCACGTA |  |  |  |  |
| Pax3 | F | GCAATGGCCTTTCACCTCAG | 57°C | 2 | ND |  |
|  | R | AGGGGAGAGAGCATAGTCGG |  |  |  |  |
| Alx1 | F | AAAGAGAACGATACGGCCAA | 54°C | 2.2 | ND |  |
|  | R | GTTCTGGATCTGTGGGTAGC |  |  |  |  |
| Alx3 | F | CATCCTCAGCTGCAGAACTC | 54°C | 2.08 | ND |  |
|  | R | GTATGGGGACATGCATGGAG |  |  |  |  |
| Ptch1 | F | GCTAGGCTAATCGTGGTTCAGTCA | 61°C | 2.05 | ND |  |
|  | R | AGCCCAACACCTGGTTCAAAGA |  |  |  |  |
| Gli1 | F | GTGTCCTCGACTTGAGCATTATGG | 61°C | 2.01 | ND |  |
|  | R | GAGCTTTGAGCTGTCCTGTGGAAT | |  |  |  |
| Gli2 | F | AGGCAAACTTTTGTCTCCTCG | 55°C | 1.9 | ND |  |
|  | R | AGACCACTCTTGGCTTCTTTCT | |  |  |  |
| Pax6 | F | TTACGAGACTGGCTCCATCA | 56°C | 2.06 | ND |  |
|  | R | ACTCCCGTTTATACTGGGCT |  |  |  |  |
|  |  |  |  |  |  |  |
|  |  |  |  |  |  |  |

ND; Not determined.

**Supplementary Table 2. Quantitative data on pregnant dams and embryos analyzed in each protocol and included in Tables 2-4.**

|  | **Treatment** | **Pregant dams (total n)** | **Live embryos/dam (mean ±SE)** | **Resorptions/dam (mean±SE)** |
| --- | --- | --- | --- | --- |
| **Table 2** | **Control** | 42 | 7.5±1.4 | 0.7±0.9 |
|  | **Vitamin E** | 30 | 8.0±1.5 | 0.7±0.7 |
| **Table 3** | **Control** | 7 | 7.6±1.8 | 1.7±1.0 |
|  | **Vitamin E** | 8 | 8.4±1.4 | 1.1±0.6 |
|  | **Vitamin E 4-9** | 7 | 8.1±2.8 | 0.6±0.8 |
| **Table 4** | **Control** | 14 | 7.5±1.5 | 1.0±0.8 |
|  | **Folate** | 21 | 7.2±2.1 | 0.6±1.0 |

**Supplementary Figures**


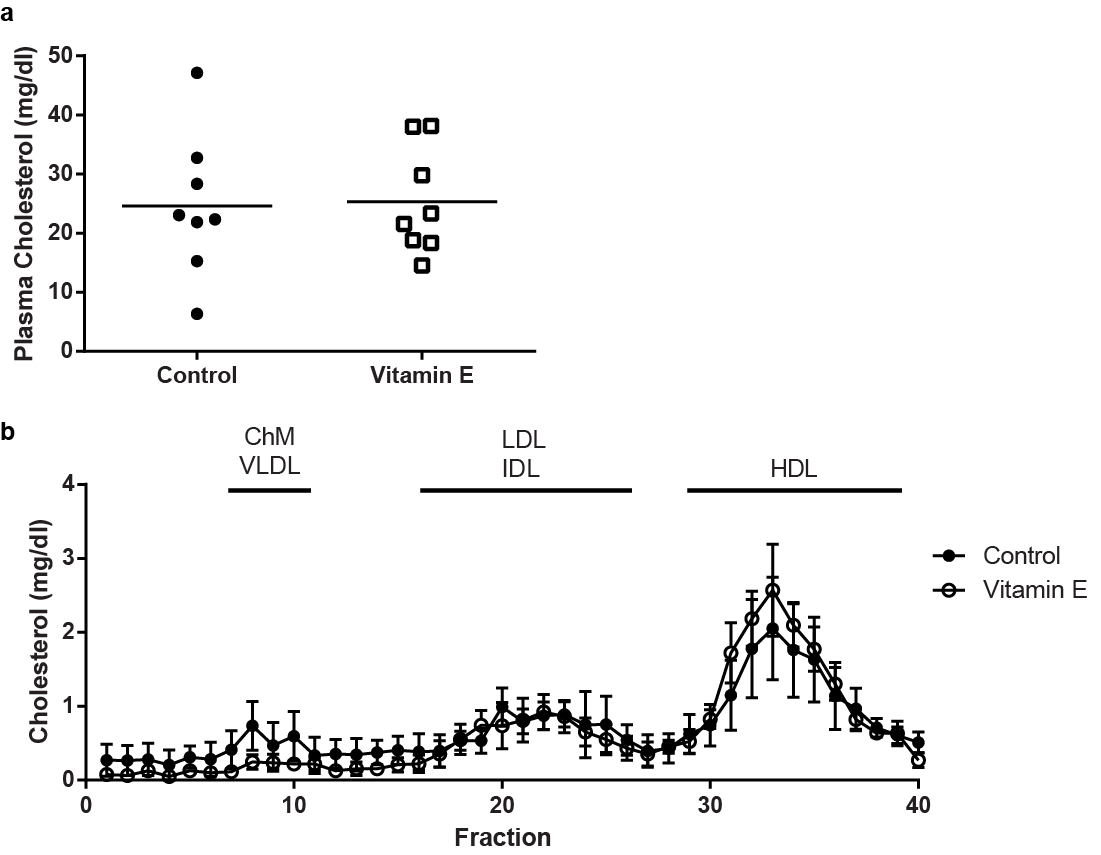


**Supplementary Figure 1. Vitamin E dietary supplementation does not modify plasmatic cholesterol in pregnant mice at E9.5.** Total plasma cholesterol concentration (a) and cholesterol distribution in different lipoprotein fractions (b) in dams fed a control or vitamin E-supplemented diets. Approximate elution positions of chylomicrons/VLDL, LDL/IDL and HDL are indicated above the elution peaks.

**
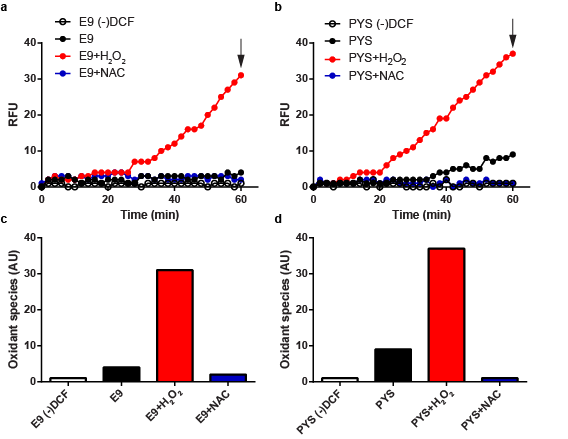
**

**Supplementary Figure 2. Representative results obtained from fluorimetry assays using embryo and PYS lysates.** Pairs of embryos or single PYS were lysed by sonication and then incubated with or without H_2_O_2_ or N-acetylcysteine (NAC) just prior to the addition of dichlorodihydrofluorescein diacetate (DCF-DA). After addition of DCF-DA, fluorescence was monitored continuously (a, b) and the value at 60 min (arrows) was used for comparison among treatments (c, d). Neither embryos nor PYS emitted fluorescence in the absence of DCF-DA [E9 or PYS (-)DCF, *white bars*] whereas fluorescence increased steadily after the addition of H_2_O_2_ [E9 or PYS +H_2_O_2_, *red bars*]. This rise was reduced by incubation of H_2_O_2_ samples with NAC [E9 or PYS +NAC, *blue bars*].


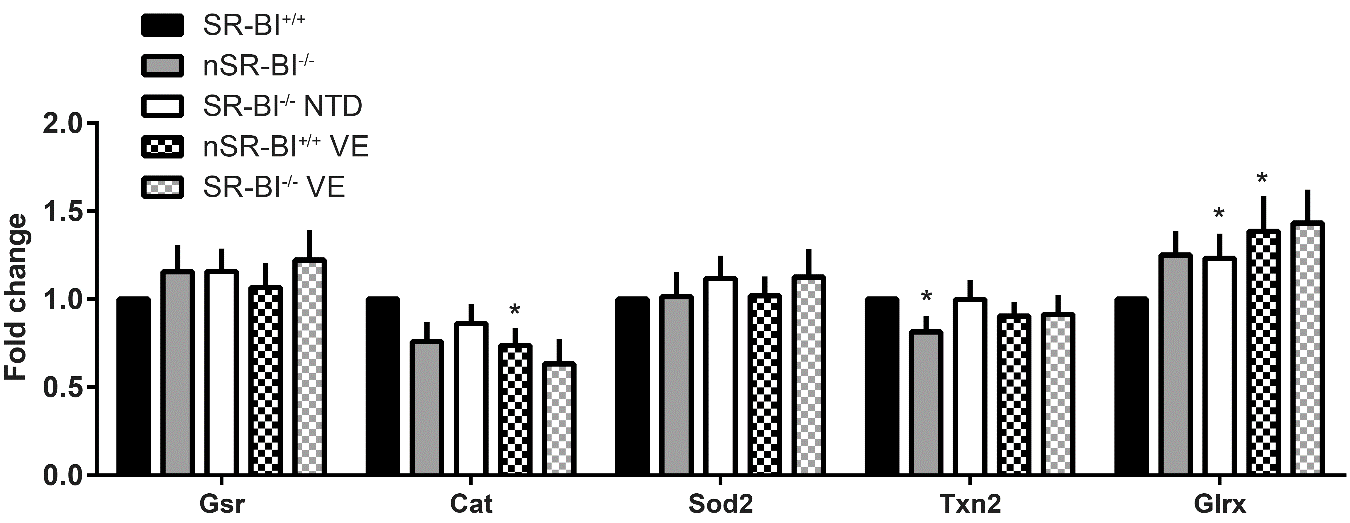


**Supplementary Figure 3. The antioxidant response is largely unchanged in PYS from SR-BI^-/-^ embryos.** Expression levels of genes involved in the antioxidant response in single PYS. Small differences with unclear biological meaning were observed. *p≤0.05 vs. SR-BI^+/+^. N=3.


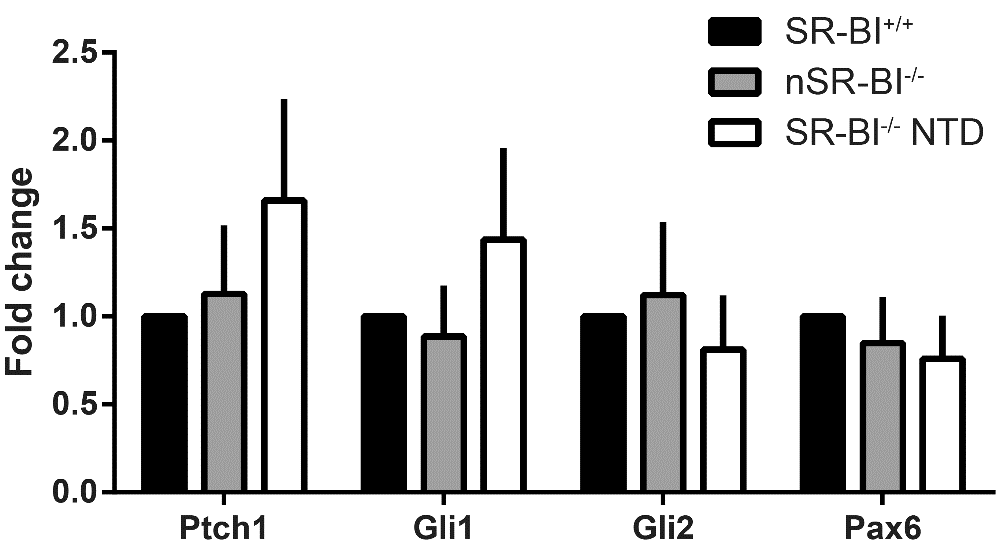


**Supplementary Figure 4. Expression of genes from the Hh signaling pathway is normal in SR-BI^-/-^ embryos.** Relative expression levels of genes directly regulated by Hh in the neural tube, measured in pools of three embryos. N=3.
